# Teams in a New Era: Some Considerations and Implications

**DOI:** 10.3389/fpsyg.2019.01006

**Published:** 2019-05-09

**Authors:** Lauren E. Benishek, Elizabeth H. Lazzara

**Affiliations:** ^1^Johns Hopkins School of Medicine, Armstrong Institute for Patient Safety and Quality, Baltimore, MD, United States; ^2^Department of Human Factors and Behavioral Neurobiology, Embry–Riddle Aeronautical University, Daytona Beach, FL, United States

**Keywords:** teams and groups, teamwork, team performance, team dynamics, team membership, team interdependence, team goals, team context

## Abstract

Teams have been a ubiquitous structure for conducting work and business for most of human history. However, today’s organizations are markedly different than those of previous generations. The explosion of innovative ideas and novel technologies mandate changes in job descriptions, roles, responsibilities, and how employees interact and collaborate. These advances have heralded a new era for teams and teamwork in which previous teams research and practice may not be fully appropriate for meeting current requirements and demands. In this article, we describe how teams have been historically defined, unpacking five important characteristics of teams, including membership, interdependence, shared goals, dynamics, and an organizationally bounded context, and relating how these characteristics have been addressed in the past and how they are changing in the present. We then articulate the implications these changes have on how we study teams moving forward by offering specific research questions.

## Introduction

Today’s organizations are markedly different than previously established. With the explosion of innovative ideas and novel technologies, organizations are redesigning the way work is accomplished (Wageman et al., 2012). This new redesign is mandating a change in job descriptions, roles, and responsibilities as well as how employees interact and perform collaborative work. According to Graesser et al. (2018), collaborative work can have potential disadvantages: ineffective communication, social loafing, diffusion of responsibility, and conflict. When harnessed correctly, though, collaborative work can entail division of labor, multiple perspectives, emergent ideas, and multi-source evaluation which enhances quality (Graesser et al., 2018). Collaborative work, as the name would suggest, involves collaborations. Collaborations manifest differently with the rise of geographic dispersion, working remotely, and collaborative technologies. Essentially, collaborations entail teams and teamwork that have evolved and resemble a new era.

The original conceptualization of teams considered them to be intact, tightly bounded, and coupled with members from a single organization who are co-located, interacting face-to-face to generate an identifiable product, service, or solution (Hackman, 2012; Tannenbaum et al., 2012; Wageman et al., 2012). Conversely, teams today consist of members from multiple organizations shifting in and out of the team while relying heavily on technology to complete a variety of tasks (Hackman, 2012; Tannenbaum et al., 2012). To illustrate, previous research has found that up to 84% of teams experience change (Espinosa et al., 2012), and another study found that the number of members from different countries was the same compared to the number of members located in the same room (Cummings and Haas, 2012). These studies simply illustrate that the archetype of teams is changing, and fluidity is increasingly prevalent.

Organizations are relying on fluid teams for several reasons. One, organizations must remain agile, responding quickly to opportunities and market changes, and strive for strategic and operational innovation (Tannenbaum et al., 2012; Chiu et al., 2017). Two, organizations are often using independent contractors to execute work, which traditionally entails a finite duration and potentially limited involvement (Chiu et al., 2017). Three, organizations rely on such teams to stimulate and energize members (Mortensen and Haas, 2016). Fourth, organizations are designing teams according to the specific skills and expertise needed to execute particular tasks, and the requisite knowledge and skills may vary as the tasks fluctuate (Tannenbaum et al., 2012).

Because organizations have new demands that leverage fluid teams, the implicit assumptions surrounding teams are not necessarily applicable. Previously, the well-established definitions assumed that the long-standing characteristics of teams (i.e., multiple members interacting dynamically and interdependently working in a bounded context toward a shared goal; Hackman, 1987; Salas et al., 1992; Cohen and Bailey, 1997; Kozlowski et al., 1999) remain stable and consistent throughout the team’s life span (Tannenbaum et al., 2012). See Table 1 for a list team characteristics highlighted in various definitions of teamwork. These characteristics, though, as originally conceptualized may not be accurate as the landscape of organizations, work, and teams has evolved considerably to be much more diverse and heterogeneous (Harrison and Humphrey, 2010; Mathieu et al., 2017).

**Table 1:** Team characteristics.

Source	Two or more members	Inter-dependent	Shared goal	Social interactions	Distinct roles	Embedded in larger entity
Alderfer, 1977	x	x			x	
Anderson and West, 1998				x		
Cannon-Bowers and Bowers, 2011		x	x		x	x
Cohen and Bailey, 1997	x	x	x		x	x
Devine et al., 1999	x		x	x		
Francis and Young, 1970	x		x			
Gladstein, 1984	x	x	x			
Guzzo and Dickson, 1996	x	x		x		x
Hackman, 1990		x			x	
Hollenbeck et al., 1995		x	x			
Katzenbach and Smith, 1998				x		x
Kazemak and Albert, 1988		x	x			
Kozlowski et al., 1999	x	x	x			x
Kozlowski and Ilgen, 2006	x	x	x	x	x	x
Kozlowski and Bell, 2003		x	x	x		x
Lanza, 1985		x	x			
McGrath et al., 2000			x			
Rasmussen and Jeppesen, 2006	x	x				
Richardson, 2010	x	x	x	x	x	x
Rousseau et al., 2006	x	x	x			
Salas et al., 1992	x	x	x			
Salas et al., 2007	x	x	x	x		
Salas et al., 2005	x	x	x			
Schippers et al., 2007		x		x		x
Shea and Guzzo, 1987	x					x
Sundstrom et al., 1990		x	x		x	x
Wageman et al., 2005		x	x			
West, 2004		x	x		x	x
West et al., 1998			x		x	x
Zander, 1977	x	x				

Understanding there is a need to discern teams differently, researchers have argued that there is a notable distinction with teams either being “real” or “pseudo” (Hackman, 2002; Wageman et al., 2005; Richardson, 2010; West and Lyubovnikova, 2012). Hackman (2002) suggested that real teams consist of four primary elements: clear boundaries, established interdependence, moderately stable members, and authority. Wageman et al. (2005) contended that real teams are comprised of three features: clear boundaries, collective responsibility for shared goals, and moderate membership stability. A more recent update posited that real teams are hallmarked by six dimensions: tightly coupled interdependence, agreed upon objectives, systematic reflex or review of performance, clear boundaries, high autonomy, and specified roles (Richardson, 2010; West and Lyubovnikova, 2012). Pseudo teams, on the other hand, are defined as a group of people who call themselves a team and work independently or interdependently toward a potentially different perception of their goal while having permeable boundaries (Richardson, 2010; West and Lyubovnikova, 2012). While we understand the desire for a distinction and applaud those trying to more aptly apprehend and examine teams, we contend that this division may not be totally suitable. The idealized conceptualization of teams is a rare reality; rather, most teams are messy. That is, most teams in today’s climate are emergent social systems that are fluid in various aspects (Chiu et al., 2017). With this fluidity in mind, it begs the question – how much variation and fluctuation in a team’s core characteristics is permissible? And, what are the implications of these characteristics with regard to team composition, process, and performance?

Recognizing the reality of teams, researchers are beginning to advocate for more novel yet realistic approaches to theorizing and investigating teams (Harrison and Humphrey, 2010; Hackman, 2012; Tannenbaum et al., 2012). Some have proposed that the concept of teams should be modified to reflect “teaming” – a continual process where teams are constituted and reconstituted (Edmondson, 2012). Others have suggested the idea of team fluidity to address this evolution of teams. However, many define team fluidity as simply changes in team membership (e.g., Dineen and Noe, 2003; Bushe and Chu, 2011). More recently, though, researchers contend that team fluidity is more than membership change because that does not accurately depict today’s teams and experiences (Chiu et al., 2017). Understanding that teams are in a new era, the purpose of this paper is to dissect each of the fundamental components of teams – membership, dynamics, interdependence, goals, and boundaries, – delineate the implications of how these components are conceptualized, and recommend avenues for future research that will better capture the current nature of team membership, contexts, and dynamics.

## Considerations Regarding the Core Characteristics of Teams

The fluidity and versatility of how actual teams operate in real-world settings present serious challenges for those scientists and practitioners attempting to understand teams. We suggest that placing careful limits and boundaries on how we qualify ‘real’ teams is not the path forward if we are to provide research insights applicable to these real-world teams. Instead, we suggest we may find more practical direction in a comprehensive/integrated deconstruction of the defining features of teams that seriously considers how fluctuations within each feature practically affect our approach to studying and improving teams.

### Membership

Perhaps the most defining characteristic of teams is membership. After all, what makes a team recognizable as a specific team is its members. Extending even further, team composition, team size, and team tenure have team membership as the foundation. According to many definitions, teams must be comprised of two or more members (Kozlowski et al., 1999; Salas et al., 2005; Kozlowski and Ilgen, 2006; Rousseau et al., 2006; Salas et al., 2007). Although these definitions do not imply that the members have to remain the same, it is often assumed that they are consistent (Wageman et al., 2012), and research has traditionally treated teams as stable entities (Hirst, 2009).

Teams that have stable membership are considered to be intact or closed (Ziller, 1965); meanwhile, teams that have fluctuating membership are thought to be open (Ziller, 1965) or fluid (Bushe and Chu, 2011), and membership changes with regards to addition, subtraction, or substitution. Bedwell et al. (2012) ascribes this fluidity to three scenarios: (1) integrating a new member to an existing team, (2) losing a member of an existing team without replacing the lost member, or (3) losing a member and integrating a new member to an existing team. Such changes can occur at the simple level of a single member to the complex level of an entire cohort (Mathieu et al., 2014). Consequently, team membership can range from “frozen rigidity” to “radical discontinuity” (Arrow and McGrath, 1995) with changes in frequency (i.e., turnover) and duration (i.e., tenure) serving as two major indices (Chiu et al., 2017). Within an organization, such membership change occurs for seven reasons: (1) desire to have different skills through various stages of work, (2) need for flexible allocation of personnel, (3) drive to provide developmental career opportunities, (4) response to high turnover, (5) need for organizational upsizing or downsizing, (6) desire to promote effective communication, and (7) motive to avoid collusive behaviors among employees (Bushe and Chu, 2011). In addition to within organization, membership change occurs across organizations. The philosophy of maintaining the same employment and retiring from the same organization is becoming an old adage (Landrum, 2017), and recent evidence suggests that ‘job hopping’ is on the rise (Robert Half, 2018).

Adding another layer of complexity to membership change is the consideration of ‘multi-team’ or multiple team membership (MTM), which refers to members serving on multiple teams simultaneously (van de Brake et al., 2018). MTM adds complexity in that there are two relevant considerations: context switching and temporal misalignment (O’Leary et al., 2011). Context switching occurs when members shift their focus from one team context to another, and temporal misalignment occurs when there is a gap in time from focusing on tasks. Understanding these considerations is important since some estimates indicate 81% of individuals have MTM (O’Leary et al., 2011), and others suggest 94.9% of members serving on multiple teams (Martin and Bal, 2006). The pervasiveness of MTMs is due to particularly skilled individuals being a desired commodity, teams being project-centered that necessitate individuals with specialized expertise, and work that has shifted toward being flat and dispersed (O’Leary et al., 2011).

Recognizing the prevalence of MTM or even membership change within a single team, researchers have begun to investigate the potential implications for taskwork, teamwork, and team performance. Two opposing views regarding the role of membership change have emerged. One school of thought frames such changes as being disadvantageous. Membership change results in a loss in individual knowledge and shared knowledge, a diverted focus away from the task, lowered member commitment, and lack of cohesion (Bushe and Chu, 2011; Bedwell et al., 2012) as well as diminished coordination (Summers et al., 2012) and reduced cooperation (Arrow and Crosson, 2003). Additionally, such teams have poorly developed shared mental models and transactive memory systems making them unable to orient quickly to new tasks and transitions (Bush et al., 2018). Finally, there is evidence that demonstrates member instability detrimentally impacts performance (Argote et al., 1995; Lewis et al., 2007); while, team familiarity strengthens team processes and states (Mathieu et al., 2014). The other school of thought frames membership change as beneficial, citing as evidence an increase in breadth of knowledge (Bedwell et al., 2012), transfer of knowledge and resources (Tannenbaum et al., 2012), and the number and diversity of ideas generated (Choi and Thompson, 2005) as well as increased productivity (Choi and Thompson, 2005) and heightened team learning (Savelsbergh et al., 2015). Moreover, such fluidity may help maintain a team’s flexibility, which is particularly beneficial in emergent situations and circumstances (Tannenbaum et al., 2012). To illustrate, teams that are more fluid may be equipped to address task conflict, which can be a beneficial catalyst for communication as well as a tool for mitigating groupthink (Bush et al., 2018). Therefore, the objective of team membership decisions should be to strategically support the organizational mission and promote organizational flexibility in competitive environments (Bell et al., 2018b). Regardless of the school of thought, membership change undoubtedly has an impact on processes, states, and outcomes.

Given that research has repeatedly indicated that a team’s members substantially influence teamwork (Mathieu et al., 2017; Bell et al., 2018a) and performance (Bell, 2007) and the ever-changing nature of membership, new questions start to surface. If membership is so fluid, how should measurement be implemented to accurately reflect the state of the team as well as the dynamism of the team? Is resorting to traditional cross section or correlational designs still appropriate? Can we make fair comparisons longitudinally if the composition of the team is different? These questions simply scratch the surface as the dynamism of membership does not only affect the team dynamics, but it also influences the team’s interdependence, goals, and boundaries. Additionally, such fluid membership also raises questions about selection, interventions, and work design that merit investigation.

### Interdependence

Interdependence refers to the level or sequencing of interaction required of team members in order to complete a given task or achieve a particular goal or outcome and is often the reason why teams are formed in the first place (Campion et al., 1993). The nature of what a team is trying to accomplish can be characterized by a two-dimensional framework – scope and complexity (Mathieu et al., 2017). A team’s objectives work symbiotically with interdependence. Interdependence moderates the relationship between team processes (i.e., cognitions and behaviors) and team performance (Gully et al., 1995, 2002; Beal et al., 2003). As such, it is a critical team feature that is almost ubiquitously included in every team definition. The underlying tenet of interdependence is that the more interdependent team members are with one another, the closer they approach “real” team status while lower interdependence is more indicative of a “working group” as opposed to a “real” team (Katzenbach and Smith, 1993).

The source of interdependence can be multifaceted. It may be determined by the nature of the task, the manner in which goals are defined, the process through which those goals are achieved, and the method for assessing team performance (Wageman, 1995; Campion et al., 1996; Van der vegt et al., 2001). *Task interdependence* refers to the degree of task-driven interaction among team members (Shea and Guzzo, 1987). Stated differently, task interdependence is the level to which colleagues must rely on one another in order to effectively perform their individual roles and job responsibilities (Saavedra et al., 1993). As task interdependence increases, demands for coordination, communication, and cooperation also tend to increase. Consistent with the idea that interdependence exists in degrees, task interdependence has been conceptualized as existing in different forms that range from a lower degree of integration to a much higher and more complex degree.

*Pooled* interdependence can be summarized as a performance-sum relationship where each member contributes to the group without needing to directly interact with other group members. Naturally, this is the lowest level of interdependence because it simply means that team performance is the simple sum of each individual’s performance. When task interdependence is pooled each team member contributes his/her own work to the final product without being reliant on any other member. A loose example of pooled interdependence might be an edited textbook wherein each chapter an author contributes content based on his or her own expertise without needing to consult the authors of other chapters. The final publication is the result of multiple authors’ contributions and would not have been possible without each, but the chapters within are individual products. *Sequential* task interdependence occurs when one group member must complete his task before another member is able to complete hers and different parts of the task must be completed in a prescribed order. The classic example is of a car assembly line where each employee performs a specific action that contributes to the final product. In this example, interdependence is a bit stronger than in the pooled example because members are dependent on others to complete their work. *Reciprocal* interdependence is the next conceptualization and occurs when team performance requires individuals to hand tasks back-and-forth between one another. These “temporally lagged, two-way interactions” (Saavedra et al., 1993, p. 63) generally exist when team members have different specialty roles that can be completed in a flexible order. For instance, two colleagues co-authoring a paper may write different sections and then go back and edit one another’s work until the manuscript is ready for review. Finally, *team* interdependence exists when members jointly diagnose, problem solve, and collaborate to complete a task. There is considerable freedom within this level of interdependence to design your own job responsibilities, but the final product requires mutual interaction. An example may be a design team working together to co-create a redesign of a gaming platform.

The problem with conceptualizing interdependence in this way is that it is probably more complex in reality than how it is presently conceived. In fact, many modern teams are involved with multiple tasks simultaneously, and each of these tasks might be associated with different levels of collaboration (Bell et al., 2018a). Similarly, the longer the lifespan of the team, the more likely it is that a work group moves between different levels of interdependence. A development team, for example, may demonstrate high-levels of interdependence when they are steeped in the divergent and convergent thinking stages of development, but as they navigate other stages of creation, they may find that their interdependencies become less complex. Furthermore, this team may find that they regularly shift between interdependencies as they come together for intense brainstorming and co-creation then somewhat disband to work on individual tasks then come together again to assemble and test their prototype.

The questions we as researchers must ask is, if interdependence is an organic moving target, how does that affect our definition and conceptualization around ‘team’? Can ‘real’ teams be considered teams if the level of their interdependencies changes over their life span? Does the shift in interdependency within a working group affect the ‘teamness’ of that group? Can a single team be more or less of a team throughout its lifespan as its interdependent nature fluctuates? Importantly, what does that mean for teams operating in the real world?

If we conclude that teams can and do in fact fluctuate with regards to interdependencies and this affects their ‘team status,’ there are clear implications for research. Measurement becomes more challenging because we will need to consider what level or even combination of interdependence teams are experiencing at the time of measurement, and if their interdependence profile is different across measurement timepoints, we may need determine whether fair comparisons can be drawn or whether we need to develop more sophisticated methods to understand the impact on their performance. We must also consider more practical concerns such as how do we make sure that team members are selected and/or trained to be able to navigate these fluctuations. It is quite possible that the team member who operates best when interdependence more closely reflects pooled or sequential process will find periods of more intense interdependence difficult to maneuver and vice versa. Thus, we will need to find better ways to support employees as they engage in various forms of collaboration.

### Goals/Shared Responsibility for Outcomes

Another defining feature of teams is the existence of at least one shared goal. This feature is central because without a shared objective, there would be no reason for multiple individuals to collaborate. They would instead be engaged in separate pursuits. However, once two or more individuals are united in the attainment of the *same* objective, they become interconnected. While the pathway to goal attainment can vary (see task interdependence), the unity between them manifests as goal interdependence and guides their performance (Saavedra et al., 1993). Thus, goals direct the attention, effort, and persistence of group members (LePine, 2005) while also influencing interactions within teams (Hu and Liden, 2011). Specifically, goals direct teams on how to define individual responsibilities, coordinate actions, and develop efficient work procedures (Klein and Mulvey, 1995). This influence manifests through planning, cooperation, mutual support, and member interactions (Mitchell and Silver, 1990; Weingart, 1992; Weldon and Weingart, 1993; Crown and Rosse, 1995).

The effort extended by group members in the pursuit of shared goals creates variance in the rewards, punishments, and feedback teams receive. Competitive and individual distribution of outcomes can inhibit team effectiveness through blocking, undermining, and hindering behaviors (Miller and Hamblin, 1963). Alternatively, shared goals can create shared responsibility for outcomes among team members (Shea and Guzzo, 1987) which are likely to enhance effectiveness by motivating members to cooperate and assist in the performance of other members (Gully et al., 2002).

The interplay of shared goals and responsibilities of outcomes clearly has implications for how teams perform. They affect team motivations, work distribution, and team member interactions because they set the direction in which the team is moving and serve as glue cementing the team together. Thus, teams are partially defined by the goal(s) they are harmonized in striving toward. The implication is that if team goals change, then the team may also be qualitatively different. Work may need to be restructured. Team members may need to be subtracted or added. The dynamics of work and team member interactions may be substantively different. In sum, the morphing of team goals and responsibilities for outcomes should be considered as a parameter for determining whether a work unit can be meaningfully compared over time or across performance contexts. Changes in team processes and emergent states may not be the result of team learning, for example, so much as they may be natural reactions to changes in goals. From a practical standpoint, the implications may be that lessons team members learned by working with one another toward different objectives may not entirely translate into the current project. Teams may discover growing pains resulting from goal shifts. It may require additional work for teams to readjust to changing demands and goals, so special efforts may be necessary as a team strives toward a new goal, even when there is a history of collaboration among its members.

Of course, from a theoretical and research perspective it means that we may have to be cautious about how we define and measure teams. If goals are substantially changing and work flows are also changing as a result, it may no longer be appropriate to consider a specific collection of individuals to be the same team. In that instance, we have to be careful about the inferences we are drawing from assessment of these groups over time or across circumstances.

### Team Dynamics

Clearly, team members must interact with one another in order to pursue shared goals and manage task interdependencies. However, team dynamics and interactions vary greatly and are moderated by a number of attitudes and behaviors. The leading taxonomy for characterizing team interactions and dynamics comes from Marks et al. (2001, p. 357) who describe processes and emergent states. *Processes* are “members’ interdependent acts that convert inputs to outcomes through cognitive, verbal, and behavioral activities directed toward organizing taskwork to achieve collective goals.” More simply, team process is the interaction of members with each other and their task environment. Processes are the means through which team members use essential and varied resources such as experience, expertise, equipment, and financial support to garner team outcomes. Thus, it is team process (i.e., action and interaction) that drives accomplishment of team goals (LePine et al., 2008).

Of course, teamwork involves more than simple behaviorally based action. Teamwork also consists of attitudes, values, cognitions, and motivations (Morgan et al., 1993; Salas et al., 2011). Marks et al. (2001, p. 357) call these affective and cognitively oriented qualities of teamwork emergent states. *Emergent states* are “constructs that characterize properties of the team that are typically dynamic in nature and vary as a function of team context, inputs, processes, and outcomes.” These team properties are states in that their quality is not guaranteed to be stable. As such, emergent states can influence how team process unfolds while themselves changing in response to team member interactions. For example, teams low in psychological safety (an emergent state) may struggle to ask each other for task assistance (a process), which might result in performance delays or errors, which could stir conflict or discord (another emergent state) within the team where none previously existed. In other words, emergent states are products of the team experience that also can impact the way in which team members interact, be it positive or negatively.

Certain competencies are needed to manage these evolving processes and dynamic emergent states. According to Cannon-Bowers et al. (1995), teamwork-related competencies vary on two domains – task and team – that span a continuum of specificity that ranges from specific to general. Task specific competencies are those that are applicable only in a specific task or type of task. For example, aviation skills are highly task-specific. Task generic competencies are those that are applicable across a variety of task settings. For instance, project management skills are applicable across a variety of projects and contexts. Correspondingly, team competencies can also be categorized as specific or general. Team specific competencies require the team members to know one another well and have experience working together whereas team generic competencies are applicable across different teams with different team members.

When considered on a matrix (see Table 2) these domains combine into four distinct types of competencies: Context-driven, task-contingent, team-contingent, and transportable. *Context-driven* competencies are specific to both the task and the team, making them highly specialized. As such, these competencies are generally best developed within in-tact teams trained or practiced in realistic settings. They are not especially good candidates for selection since it is difficult to understand in advance how a team member may integrate his or her KSAs into an existing team. *Task-contingent* competencies are specific to the task but not to the team. These are best trained in a realistic task environment and may be useful for selecting new team members. Team-contingent competencies are specific to the team but not to the task. It is generally unhelpful to select team members based on these competencies and instead they are better developed with intact teams across a variety of tasks. Finally, transportable skills are the most flexible. They are applicable to all teams across all tasks.

**Table 2:** Matrix of teamwork competencies.

		Team	
		Specific	Generic
**Task**	**Specific**	Context-driven	Task-contingent
	**Generic**	Team-contingent	Transportable

Irrespective of the specifics of the task or team, all teams experience transitions as they evolve from one task to the next. Marks et al. (2001) clearly outlined the relevant processes for transition periods (i.e., mission analysis, goal specification, and strategy formulation) as well as the processes for action periods (i.e., monitoring goal progress, systems monitoring, team monitoring, and coordination); however, modern teams likely do not experience these clear delineations as postulated by Marks et al. (2001). In other words, modern teams experience transitions along a continuum of length and punctuated between tasks that range on a continuum from similar to dissimilar (Bush et al., 2018). Consequently, the requisite processes may differ given the temporality of the transition periods and the similarity (or dissimilarity) of the tasks surrounding those transition periods.

As teams maneuver these phases, they must make decisions in an evolving world, requiring them to be flexible in the presence of change. Team adaptation is the process through which teams respond cognitively, affectively, and behaviorally to change (Baard et al., 2014), which can stem from internal (e.g., membership turnover) or external (resource availability) sources (Frick et al., 2018). Successful adaptation has beneficial outcomes for teams; however, it may also manifest maladaptively for numerous reasons (Frick et al., 2018). Frick et al. (2018) describe the Four Rs heuristic to explain how team adaptation occurs and explain the points of failure in this process that could result in maladaptation. The stages include recognize (i.e., noticing and acknowledging a change), reframe (i.e., shifting cognitions about the situation as a result of the change), respond (modifying behavior), and reflect (i.e., contemplating the change and the team’s subsequent response).

Affecting these tasks and transitions while constraining and influencing team dynamics is team structure. Team structure encompasses the team relationships that drive the assignment of tasks, roles and responsibilities, and leadership (Bresman and Zellmer-Bruhn, 2013; Chiu et al., 2017). Like many other defining elements of teams, structure has historically been assumed to be somewhat stable in nature. That is, task assignments are pre-defined, roles and responsibilities are clear and consistent over time, and leadership manifests as command and control (Chiu et al., 2017). However, in modern teams these traits are also increasingly fluid. Task assignments occur on an as-needed basis and are given to team members with the ability and bandwidth to perform them. Roles and responsibilities, therefore, become more blurred (Dube, 2014) as team members coordinate to move with greater adaptability and agility. Leadership is increasingly self-directed (Aime et al., 2014) and shared across team members (Carson et al., 2007). Team member status emerges quickly based on observable characteristics and expected performance but is subject to change if those in positions of authority fail to perform adequately (Driskell T. et al., 2018). The result is that modern teams rely less on stringent pre-defined plans, rules, procedures, and communication norms (Malone and Crowston, 1994) and more on informal and emergent coordination (Okhuysen and Bechky, 2009).

While these frameworks help us to organize the way we approach, think about, and manage team dynamics, they somewhat fail to account for the complexity that are real world teams. For example, it is likely that many teams require some combination of specific, general, team, and task competencies to support team emergent states and processes. These competencies are further influenced by the transitions teams experience between tasks. In fact, even the dynamics themselves as well as the transitions may be contingent upon the tasks.

### Team Boundaries

The final defining characteristic of a team is the idea that a team does not exist in a vacuum but rather is influenced by context. According to Bell et al. (2018a), context shapes the team in three ways. One, the context influences the salience of a particular attribute. Two, the context can alter the relevance and importance of an attribute. Three, the context ignites which attributes are of value. Some conceptualize context broadly by making a distinction between external, influences mostly outside of the control of the team, and internal, influences within the team (Bell et al., 2018a). Meanwhile, others conceptualize context more granularly by referring to context as the characteristics of the task, the timeframe of the performance episode(s), the governance structure over the team, and a team being embedded within a larger entity or context (Edmonson and Harvey, 2018). In essence, the context functions by providing boundaries.

Boundaries in a general sense facilitate togetherness and serve as a distinction between what something is versus what it is not (Alderfer, 1976). Within the team context, a team has boundedness with boundedness being a delineation between members and non-members, and individuals use three criteria to identify boundedness (Mortensen and Haas, 2016). One, members rely on an official team roster (formal criterion). Two, individuals receive the label of team member by themselves or someone else (identity-based criterion). Three, members are identified through a pattern of interactions (interaction-based criterion). Although these criteria may provide clarity for how boundedness is determined, and literature often assumes clear team boundaries are the norm, the actual boundaries in real-world teams are often less clear (Tannenbaum et al., 2012). Some have long proposed that boundedness may be actually be a spectrum with highly permeable boundaries (i.e., underbounded) to highly impermeable boundaries (i.e., overbounded; Alderfer, 1980); meanwhile, others have posited that boundaries are more dynamic and fluid and are constituted and reconstituted (Edmondson, 2012). In fact, there is so much fluctuation that it is often difficult to determine who comprises the team (Hackman, 2012).

Such ambiguous boundaries are a result of team fluidity, overlap, and dispersion (Mortensen and Haas, 2016). Fluidity entails members who are dynamically moving in and out of the team. Overlap involves members who work on multiple teams simultaneously, and dispersion refers to members working from different organizations or geographic regions. Such fluctuations in team membership are often arranged and coordinated rather than being chaotic and impromptu (Tannenbaum et al., 2012). Because the boundary is being reshaped with such fluctuations, it impacts shared identity and shared understanding. With every fluctuation, the team must rebuild its identity and must update the shared understanding based upon the member’s mental models of the team, task, and context.

Fluidity, overlap, and dispersion affects boundedness between the team and the outside context, but it also affects boundaries *within* the team and the tasks. Team members create boundaries within the team based upon the extent that they perceive themselves to be similar to one another. That is, team members rely upon surface-level cues (i.e., attributes that are easily accessible and detectable) and deep-level cues (i.e., psychological characteristics) to inform categorization. Categorization enables team members to rely upon heuristics which can serve as an impetus for subsequent attitudes and behaviors (Feitosa et al., 2018). Consequently, such categorization and perceptions influence the roles, interactions, and structures (Bell et al., 2018a; Feitosa et al., 2018; Graesser et al., 2018). To elaborate, teams often develop a core and a periphery structure (Tannenbaum et al., 2012; Mortensen and Haas, 2016). Albeit colloquially, the concept of a core and a periphery is analogous to an inner and outer circle. The core structure is comprised of members who perform a “major” role; whereas, the periphery structure includes members who perform a more “minor” role. Similarly, tasks can also manifest as a central working sphere and a peripheral working sphere (Gonzalez and Mark, 2004). A central working sphere is considered important and urgent; whereas, a peripheral working sphere is deemed to be less important and critical. Additionally, members dedicate more time on central working spheres yet allocate minimal time toward peripheral working spheres.

Given the dynamism of boundedness between entities, within teams, and tasks, it is evident that boundary clarity is integral. When teams experience boundary clarity, members experience individual certainty, and the team experiences a collective agreement (Mortensen and Haas, 2016). Conversely, teams that have poor boundary clarity are comprised of members with individual uncertainty and an overall sense of collective disagreement. Members are unsure of who is considered a member of the team, and members have opposing views on who is an actual member of the team.

Regardless of the clarity or ambiguity, the boundedness of a team has implications for researchers and practitioners (Mortensen and Haas, 2016). That is, researchers may need to alter their theorizing and measuring depending upon the stability and clarity of the boundedness. For example, many team processes or states are grounded in the idea that teams are tightly coupled and bounded (e.g., transactive memory systems), but how do these manifest if teams have loose and permeable boundaries? Similarly, roles and responsibilities are often theorized based on the assumption that they remain consistent, but if a team’s boundaries are fuzzy, the idea of a boundary spanner needs revisiting (Mortensen and Haas, 2016). Practitioners, similarly, may need to select, design, and support teams differently depending upon the consistency and certainty of the boundedness.

## Implications

For decades, the needs and experiences that teams faced in the real-world as well as the policies and procedures that practitioners used to manage teams corresponded to the studies that researchers were conducting (Tannenbaum et al., 2012; Wageman et al., 2012). However, the organizational landscape that has manifested is not always aligning with prevailing research; therefore, research and even practice needs to evolve according to current needs to advance team effectiveness. Although “old questions” become relevant again when the very nature of teams has changed (Wageman et al., 2012), others argue that the questions should actually shift given the gravity of changes (Mathieu et al., 2017). Below we discuss implications of the evolution of teams in the modern era for research.

### Team Types

When attempting to understand what constitutes a team, many have theorized about team types. For example, Sundstrom et al. (1990) postulated that there are four main team types: advice/involvement, production/service, action/negotiation, and project/developmental teams. Cohen and Bailey (1997) followed suit by suggesting there are project teams, traditional work teams, parallel teams, and management teams. Devine et al. (1999) created another taxonomy to include four team types: ad hoc project teams, ongoing project, ad hoc production, and ongoing production and actually modified the taxonomy to include 14 different team types (Devine, 2002). Even still, De Dreu and Weingart (2003) created their own team type taxonomy, which included project teams, production teams, decision making teams, and mixed teams. Finally, Wildman et al. (2011) presented a team type taxonomy based upon tasks: managing others, advising others, human service, negotiation, psychomotor action, defined problem solving, and ill-defined problem solving. Although these are simply several examples demonstrating various interpretations and suggestions for team type taxonomies, it does portray that there is no consensus regarding how teams should be classified and that many taxonomies approach classification based primarily on task type. Teams have greater distinctions beyond task type, so such categorization actually limits our apprehension of team effectiveness (Tannenbaum et al., 2012). Recognizing the limitations of instituting a categorical classification system for team types, Hollenbeck et al. (2012) created a dimensional scaling framework to describe teams positing that teams varied on authority differentiation, skill differentiation, and temporal stability. The dimensional scaling approach is closer to potentially representing teams; however, the theory might need to be altered further to account for the dynamism of all facets of modern teams. For example, because boundaries can be ambiguous and membership can be fluid, the team type may also change with time and as the team progresses and transitions between tasks. If the team type does in fact change and is in fact dynamic, what are the implications for teamwork and taskwork as well as team and task performance? Do variations on teams (e.g., virtual teams; Gilson et al., 2015 and multi-team systems; Shuffler and Carter, 2018) impact teamwork, taskwork, and outcomes? Essentially, what constitutes different teams may need to be updated with the changes to reflect contemporary work and organizations. Understanding what constitutes such teams as well as what conditions are most important helps lead to greater insights regarding team effectiveness (Hackman, 2012). Questions for future research include:

• What features beyond the task constitute team types? How does the evolution of these features impact the team type?
• What approaches are most suitable for characterizing teams (e.g., categorical or dimensional scaling)?
• What other categorizations or dimensional scaling factors need to be considered and included?
• How do variants on traditional team types impact teamwork?

### Models and Frameworks

Perhaps the foundation of most team theorists is the depiction of team effectiveness models. Many team models are rooted in the input–process–output (IPO) foundation put forth by McGrath (1964). Inputs are the antecedents that influence the dynamics of team members. The processes are the interactions that team members undertake to achieve the desired goal, and the outputs are the outcomes or results accomplished by the team. As Hackman (2012, p. 431) says, “the core idea of the model is that input states affect group outcomes via the interaction that takes place among members.”

Despite being a valuable infrastructure, the IPO framework has several limitations leading others to modify the original conceptualization. Many adaptations have included an environmental or contextual component since teams do not operate in a vacuum and are certainly influenced by contextual factors (e.g., Cohen and Bailey, 1997). Introducing a contextual component lead to the realization of the multilevel nature of teams – individuals are nested within teams, and teams are nested within organizations which exist within even broader environments (Klein and Kozlowski, 2000). A second limitation of the IPO approach is the narrow focus on process. Processes are interdependent cognitive, verbal, and behavioral activities that convert inputs to outputs (Marks et al., 2001), but not all teamwork components are simply processes. Teamwork is also comprised of emergent states, which are properties that represent the attitudinal and cognitive properties of the team (Marks et al., 2001). Further, not all “processes” are mediators as originally depicted in the IPO organization; unpacking teamwork entails that some processes and emergent states can be moderators as well as mediators. Understanding these conceptual limitations, Ilgen et al. (2005) delineated “process” by presenting the input–mediator/moderator–output–input (IMOI) framework. A third limitation in the IPO approach is the lack of temporality, noting the limitations of suggesting that teams operate linearly and not episodically (Marks et al., 2001). To address the temporality of teams, there are two prominent approaches: developmental and episodic (Mathieu et al., 2008). The developmental approach suggests that teams have differential influences and qualitatively change over time. The episodic approach posits that teams exhibit different processes and states at different times. See Mathieu et al. (2008) for a review of team effectiveness.

All of the models that attempt to address previous limitations certainly advance our understanding of the complex phenomena of team effectiveness; however, we argue that more work regarding the theoretical nature of teams is still needed. The influences and the underpinnings of teams do not reside in clear and distinct packages, but rather the effectiveness of teams lies in the complex web inherent within teams and teamwork (Hackman, 2012). Modern teams are likely not well-represented within simple cause-effect models because what ensues as teams strive to accomplish their goal(s) is not a linear progression; instead, a complex combination of factors varying differentially is a more accurate representation. Modern teams are likely to juggle tasks over time, experience membership churn, coordinate with other teams, and reconfigure throughout its lifecycle (Driskell J.E. et al., 2018). Future approaches should consider more sophisticated frameworks that move beyond causal models and involve an analysis of all factors and conditions (Hackman, 2012). Additionally, other processes, such as team creativity and innovation, and emergent states, such as team well-being, may become more central drivers of modern team effectiveness and should situate more prominently in team performance frameworks and research (Driskell J.E. et al., 2018). With these considerations, we put forth the following research questions:

• What approach(es) are more suitable if the input-process-state-output structure is outdated and not applicable?
• How should modern team effectiveness models and frameworks be represented to accurately depict contemporary teams?
• How can team models and frameworks correctly depict the fluidity of all of the characteristics of modern teams?
• What team attitudes, behaviors, and cognitions should be considered when creating team effectiveness frameworks?
• What are the relationships between all of the contextual factors as well as the team processes, states, and outcomes if they are not linear?
• What are the unique contributions of team attitudes, behaviors, and cognitions, and how do these vary over time?
• How can temporal components be best incorporated and depicted?

### Measurement

As we have indicated, previous thinking depicted teams with relatively stable factors (e.g., goals and roles). Because team factors were primarily theorized as being stable, they are often only measured once or used as correlates (Tannenbaum et al., 2012). Such data is often collected at the individual level, but because it is evident that individuals are nested within teams, individual data is often aggregated to the team level. Some argue, though, that simple linear aggregations are not appropriate since the inputs, processes, and states are not perceived similarly across members and are not interchangeable (Murase et al., 2012). Aggregates represent compositional characteristics, and compositional thinking assumes the content and structure are created linearly and represented similarly (Bell, 2012). The characteristics of today’s teams are much more fluid and dynamic. Therefore, teams and the factors that comprise teams need to be studied and measured with regards to patterns over time (Bell, 2012; Mortensen and Haas, 2016). More specifically, measures of patterns could include: density, reciprocity, transitivity, and centrality. Studying networks and patterns is more representative since extensive fluidity raises the question of whether the relevant team members are being measured across time and whether the multilevel nature of teams is being captured. Simply stated, researchers are at risk of comparing different sets of team factors (e.g., membership) when only using cross sectional measurement (Murase et al., 2012). A network approach acquires information about individuals and their attributes as well as the team-level properties, and it captures the nature of the interactions. This approach is useful for understanding where an individual is embedded within a larger team or multiteam system (Bell et al., 2018b), helping to identify essential players and create a more comprehensive understanding of teamwork through a relational lens. Ultimately, theorizing and researching current teams requires a shift from the old fashioned to a more modernized approach. Mathieu et al. (2017) posit that a more modernized approach likely means that there is no standard set of measures for team research. The specifics that influence or are inherent within each team vary too greatly between and across teams making them markedly different and necessitating more nuanced metrics. Consequently, we propose the following questions:

• What research designs should be leveraged to most appropriately generalize from lab settings and study teams in applied settings?
• What statistical techniques should be employed to correctly represent the complex web of teamwork and team performance?
• What approaches are more suitable if central tendencies no longer provide a clear picture and understanding?
• What tools and metrics are most suitable to best understand and unpack the simultaneous and interrelated nature of teamwork? What tools and metrics are appropriate for time-dependent constructs?
• How can illustrative case studies be leveraged to highlight novel constructs and relationships?
• What continual streaming metrics (e.g., wearable sensors) can be utilized to address longitudinal issues?

### Staffing Teams

The dynamism and fluidity of today’s teams present special challenges for staffing teams. In some cases, such as in surgical units, teams may come together for fairly brief periods of time, even just a few hours to complete a single surgery. These teams might complete multiple projects or cases in rapid succession, or they may disband after just one project together. It is possible for these short-duration teams to reconfigure, sometimes with a majority subset of the original team and other times with a composition of team members that barely resembles the original team. This is a stark difference from the “traditional” team, with its longer-duration lifecycle and mostly stable membership. Traditional teams have the benefit of time, allowing them to more deeply develop critical emergent states like trust, psychological safety, and transactive memory systems. Because members of traditional teams have to work with one another for longer durations, it is possible that individual idiosyncrasies and work habits are more important in these contexts. However, for the rapid cycle teams that are appearing with greater regularity in the modern workplace, these individual differences may be less important to staffing a team. Instead, it is likely that *who* is on the team is less important than what knowledge, skills, and attitudes they contribute. Selection, therefore, may require less attention on compatibility of team members’ personality and work preferences and instead emphasize the compatibility of team member strengths and competencies.

We must also consider how dynamism in roles, goals, and tasks can impact selection of team members. Teams should be staffed based on members’ value to organizational competitive advantage (Bell et al., 2018b). Changing needs, which may or may not be anticipated, adds complexity to the issue of staffing teams. While each team member may still bring a specific background or expertise to the team, expertise particulars may not be able to be successfully anticipated. It, therefore, may be more appropriate to look for individuals with certain attributes that might facilitate adaptability to changing circumstances and demands. Such attributes may include learning orientation, self-directed motivation, tolerance for ambiguity, and willingness to empathize, brainstorm, and prototype. It is also likely that selection should focus on identifying candidates with transportable teamwork competencies as opposed to those that are task-contingent.

As always, identifying team members for cultural fit is paramount when staffing teams, even as teams become more and more dynamic and short-lived. It is potentially even more important for staff working on rapid-cycle teams to have a strong identification with the company culture so that these individuals are better able to collaborate and coordinate with other members of the organization even as their teams assemble, disband, and reassemble in different configurations and navigate changing expectations, goals, and demands. Organizational value congruence is expected to reduce both task and relationship conflict between team members (Chuang et al., 2004), therefore, selecting staff for congruence with organizational values will help team members subject to participating on multiple teams or teams that quickly configure and disband work collaboratively with their colleagues. With this in mind, we present the following research questions:

• What competencies or values should be considered when staffing teams?
• How does staffing influence the manifestation of team competencies or values?
• How does the fluidity of team characteristics (e.g., interdependence) impact staffing decisions? Conversely, how does staffing impact the dynamism of team characteristics?
• What are the qualities of a flexible team member, and how can team members help one another to become more flexible?
• How do team member characteristics impact how work is completed?
• What task characteristics should be considered when staffing modern teams? How does the evolution of the task and its characteristics influence staffing decisions?

### Team Interventions

Of course, it does not make logistical, practical, or even conceptual sense to rely on selection as the main source of controlling team membership and performance during dynamic situations. However, team-based interventions (i.e., systematic activity aimed at strengthening team competencies and dynamics and improving team performance; Lacerenza et al., 2018) are also employed. As described above, interventions are especially useful for modifying context-specific and team-specific competencies (Cannon-Bowers et al., 1995). However, given the faster pace of change in modern organizations and the agility that many teams must demonstrate in order to perform well, traditional approaches to interventions may also need to be re-thought. Known hallmarks of well-designed training include communicating information, demonstrating the principles, skills, or behaviors to be learned, providing opportunities for students themselves to practice, and providing subsequent feedback on their performance (Salas et al., 2012, 2015). Typically, instruction has been constrained to formal classroom style approaches wherein participants come together in a face-to-face setting and learn from an instructor. In these sessions, participants usually must plan in advance to attend, register, commute to the classroom, and have protected time in their calendars to participate in training, all of which can present as barriers to the communication of necessary information. Traditional approaches may be suitable for teaching transportable competencies but may no longer be sufficient for imparting other types of knowledge, skills, and attitudes when needed. They also may not reflect how learning actually takes place. Much of learning is experiential, occurring in informal or on-the-job real-world settings (Shank, 2012).

On-the-job training is not a new idea. Adult learners want to have access to information, practice, and feedback when they need it most and since experience plays a major part in how we learn and perform, the thought of incorporating these lessons at work, where they are most relevant is attractive. Compound this proclivity for convenience and applicability of materials to current work processes with a dynamic environment and the implication is that interventions intended to improve teamwork and team performance may be better presented on-the-job, to actual team incumbents. Furthermore, with changing conditions, whether they are reconfigured team membership, a new team goal, changing interdependencies, or new task assignments and responsibilities, there is greater need to have access to relevant information and interventions real-time. Teams may not have the time or resources to schedule formal training off-site, taking time away from their jobs and the work that needs to be accomplished.

The natural next question is what delivery mechanisms can be used that would be suitable for these demands? Technology is likely to play a large role. Access to online repositories of information that teams and individual team members can access and download at will would be ideal. Of course, incorporating demonstration, practice, and feedback opportunities into these materials will be equally important and may require more creative approaches when a knowledgeable instructor, mentor, or coach is unavailable to provide teams with direction and sensemaking of content. Teams would also need access to reliable equipment like internet and computers capable of presenting content. For many teams, these materials are easily accessed but for teams such as military, medical, and construction teams that work in a variety of settings equipment of this type may not be already provided. Take construction teams as an example; these teams may not have access to reliable WiFi while on a job site. However, most Western employees do have access to smartphones and data plans. Practitioners and researchers should consider how these technologies can be tapped as a platform for accessing team resources real-time.

Finally, interventions and delivery of content for modern teams needs to be bite-sized and digestible, with only relevant information being presented. Teams operating in dynamic environments may not have the time to muddle through excessive content when there is work to be completed and very little, if any, dedicated time for additional learning. Couple that with recent estimates that the adult attention span may be as short as eight seconds (Microsoft, 2015), and there is further evidence for the need to keep intervention and informational content brief. Finding the balance between how little content is essential and how much content is excessive will be a challenge moving forward, especially when individual learner needs will naturally vary. Regarding team interventions, we propose the following research questions:

• How do we embed interventions within the team performance context so they are available precisely when needed?
• What tools and resources beyond training (whether classroom-based or on-the-job) can help teams respond flexibly to changing demands?
• How should interventions be employed given the movement toward globalization and the rapid advent of new technology?
• To whom should interventions target when attempting to maximize teamwork and team performance?

### Digitization and Technology

Perhaps the biggest factor influencing how we define, work in, and study teams is digitization. Digital technologies are radically changing the world and the ways we live and work. Face-to-face meetings have given way to phone and video conferencing; paper-based mail has been replaced by email; typewriters exchanged for laptops and smart phones; wired connections substituted for wireless always-connected devices. Team members are able to communicate with each other across time and distances in ways that were previously impossible. Tannenbaum et al. (2012) outline many of the advantages and pitfalls that technology has for teamwork. Among the perceived advantages are greater ability to collaborate over distance (enabling the collaboration of experts across the globe), automatization of routine tasks, swifter communication, and flexibility in scheduling. These characteristics have the potential to enhance teamwork and team effectiveness, but they come with their own set of challenges that may off-set potential gains. For example, it is easier than ever to work non-traditional hours. While the flexibility afforded by technology may be believed to facilitate individual employee productivity, it can also invade personal time for non-work activities and create dissatisfaction with work-life balance (Barber et al., 2019). While employees are working longer hours, this does not necessarily translate into greater productivity as they forego necessary rest and down time needed for renewal (Fritz et al., 2010). Furthermore, technology-mediated collaboration can create lags in information exchange, more misunderstandings, fewer information seeking attempts, and less coherent messages (Andres, 2012).

Digitization and technology may underlie most, if not all, of the challenges and advancements we see in modern teams. However, much like Pandora, we cannot put our digital tools back in their boxes. The world in which we collaborate is not like the world of yesterday; but neither does tomorrow’s world look like that which we see today. The technologies we are using now will likely be outdated within a decade, and teams will continue to evolve. Future teams research centered on technology and digitization might explore:

• How can technology be leveraged to facilitate increasingly important team processes, such as creativity?
• What impact does technology have on team and team member well-being?
• Does technology need to mimic the advantages of face-to-face interactions or can teamwork be organized to better leverage the advantages of technology?
• How do technologies impact the emergence of team states such as cohesion, trust, identity, and adaptation?
• How do we limit the invasiveness of technology within our collaborations?

## Conclusion

Teams have been ubiquitous, so there have been longstanding theories and research. However, teams are very different given the macro trends in organizations and tasks. Consequently, these well-established theories and methodologies may necessitate some modernizing as the landscape of teams looks very differently in today’s society. Contemporary teams and collaborations require new thinking and approaches to gain real insights and answer enlightening questions (Murase et al., 2012; Wageman et al., 2012). Additionally, as Tannenbaum et al. (2012) indicated, the need for future research is exacerbated by conflicting evidence (e.g., membership fluidity). To understand what novel thinking and research is necessary, we must first unpack the defining components of teams. Thus, the purpose of this paper was delineate how the traditional defining characteristics of teams are actually being represented in the real working environment and offer avenues for investigators to conduct future research to better unpack the theorizing and implications surrounding teams. We hope that future researchers begin to dissect the theory regarding and surrounding teams with finer detail to advance an accurate depiction of contemporary teams.

## Author Contributions

Both authors contributed substantially to the development of ideas, drafting, and preparation of the final manuscript.

## Conflict of Interest Statement

The authors declare that the research was conducted in the absence of any commercial or financial relationships that could be construed as a potential conflict of interest. The handling Editor declared a shared affiliation, though no other collaboration, with one of the authors LB.

## References

[B1] Aime F. Humphrewy S. DeRue D. S. Paul J. B. (2014). The riddle of heterarchy: power transition in cross-functional teams. *Acad. Manag. J.* 57 327–352. 10.5465/amj.2011.0756

[B2] Alderfer C. P. (1976). “Change processes in organizations,” in *Handbook of Industrial and Organizational Psychology* ed. Dunnette M. (Chicago, IL: Rand McNally) 1591–1638.

[B3] Alderfer C. P. (1977). “Group and intergroup relations,” in *Improving Life at Work* eds Hackman J. R. Suttle J. L. (Santa Monica, CA: Goodyear) 227–296.

[B4] Alderfer C. P. (1980). “Consulting to underbounded systems,” in *Advances in Experiential Social Processes* eds Alderfer C. P. Cooper C. L. (Chichester: John Wiley and Sons) 267–295.

[B5] Anderson N. R. West M. A. (1998). Measuring climate for work group innovation: development and validation of the team climate inventory. *J. Organ. Behav.* 19 235–258. 10.1002/(SICI)1099-1379(199805)19:3<235::AID-JOB837>3.0.CO;2-C

[B6] Andres H. P. (2012). Technology-mediated collaboration, shared mental model and task performance. *J. Organ. User Comput.* 24 2558–2580.

[B7] Argote L. Insko C. A. Yovetich N. Romero A. A. (1995). Group learning curves: the effects of turnover and task complexity on group performance. *J. Appl. Soc. Psychol.* 25 512–529. 10.1111/j.1559-1816.1995.tb01765.x

[B8] Arrow H. Crosson S. (2003). Musical chairs: membership dynamics in self-organized group formation. *Small Group Res.* 34 523–556. 10.1177/1046496403254585

[B9] Arrow H. McGrath J. E. (1995). Membership dynamics in groups at work: a theoretical framework. *Res. Organ. Behav.* 17 373–411.

[B10] Baard S. K. Rench T. A. Kozlowski S. W. (2014). Performance adaptation: a theoretical integration and review. *J. Manag.* 40 48–99. 10.1177/0149206313488210

[B11] Barber L. K. Conlin A. L. Santuzzi A. M. (2019). Workplace telepressure and work life balance outcomes: the role of work recovery experiences. *Stress Health* 10.1002/smi.2864 [Epub ahead of print]. 30882979

[B12] Beal D. J. Cohen R. R. Burke M. J. McLendon C. L. (2003). Cohesion and performance in groups: a meta-analytic clarification of construct relations. *J. Appl. Psychol.* 88 989–1004. 10.1037/0021-9010.88.6.989 14640811

[B13] Bedwell W. L. Ramsay S. Salas E. (2012). Helping fluid teams work: a research agenda for effective team adaptation in healthcare. *Transl. Behav. Med.* 2 504–509. 10.1007/s13142-012-0177-9 24073150 PMC3717940

[B14] Bell B. S. (2012). Three conceptual themes for future research on teams. *Ind. Organ. Psychol.* 5 45–48. 10.1111/j.1754-9434.2011.01403.x

[B15] Bell S. T. (2007). Deep-level composition variables as predictors of team performance: a meta-analysis. *J. Appl. Psychol.* 92 595–615. 10.1037/0021-9010.92.3.595 17484544

[B16] Bell S. T. Brown S. G. Colaneri A. Outland N. (2018a). Team composition and the ABCs of teamwork. *Am. Psychol.* 73 349–362. 10.1037/amp0000305 29792453

[B17] Bell S. T. Brown S. G. Weiss J. A. (2018b). A conceptual framework for leveraging team composition decisions to build human capital. *Hum. Resour. Manag. Rev.* 28 450–463. 10.1016/j.hrmr.2017.06.003

[B18] Bresman H. Zellmer-Bruhn M. (2013). The structural context of team learning: effects of organizational and team structure on internal and external learning. *Organ. Sci.* 24 1120–1139. 10.1287/orsc.1120.0783

[B19] Bush J. T. LePine J. A. Newton D. W. (2018). Teams in transition: an integrative review and synthesis of research on team task transitions and propositions for future research. *Hum. Resour. Manag. Rev.* 28 423–433. 10.1016/j.hrmr.2017.06.005

[B20] Bushe G. R. Chu A. (2011). Fluid teams: solutions to the problems of unstable team membership. *Organ. Dyn.* 40 181–188.

[B21] Campion M. A. Medsker G. J. Higgs A. C. (1993). Relations between work group characteristics and effectiveness: implications for designing effective work groups. *Pers. Psychol.* 46 823–847. 10.1111/j.1744-6570.1993.tb01571.x

[B22] Campion M. A. Papper E. M. Medsker G. J. (1996). Relations between work team characteristics and effectiveness: a replication and extension. *Pers. Psychol.* 49 429–452. 10.1111/j.1744-6570.1996.tb01806.x

[B23] Cannon-Bowers J. A. Bowers C. (2011). “Team development and functioning,” in *APA Handbooks in Psychology. APA Handbook of Industrial and Organizational Psychology* Vol. 1 *Building and developing the organization* ed. Zedeck S. (Washington, DC: American Psychological Association) 597–650.

[B24] Cannon-Bowers J. A. Tannenbaum S. I. Salas E. Volpe C. E. (1995). “Defining competencies and establishing team training requirements,” in *Team Effectiveness and Decision Making in Organizations* eds Guzzo R. Salas E. (San Francisco, CA: Jossey-Bass) 333–380.

[B25] Carson J. B. Tesluk P. E. Marrone J. A. (2007). Shared leadership in teams: an investigation of antecedent conditions and performance. *Acad. Manag. J.* 50 1217–1234. 10.5465/amj.2007.20159921

[B26] Chiu Y.-T. Khan M. S. Mirzaei M. Caudwell C. (2017). “Reconstructing the concept of team fluidity for the digitized era,” in *Paper Presented at ISPIM Innovation Summit* Melbourne.

[B27] Choi H. Thompson L. (2005). Old wine in a new bottle: impact of membership change on group creativity. *Organ. Behav. Hum. Dec. Process.* 98 121–132. 10.1016/j.obhdp.2005.06.003

[B28] Chuang Y. Church R. Zikic J. (2004). Organizational culture, group diversity and intra-group conflict. *Team Perform. Manag.* 10 26–34. 10.1108/13527590410527568

[B29] Cohen S. G. Bailey D. E. (1997). What makes teams work: group effectiveness research from the shop floor to the executive suite. *J. Manag.* 23 239–290. 10.1016/s0149-2063(97)90034-9

[B30] Crown D. F. Rosse J. G. (1995). Yours, mine, and ours: facilitating group productivity through the integration of individual group goals. *Organ. Behav. Hum. Dec. Process.* 64 138–150. 10.1006/obhd.1995.1096

[B31] Cummings J. Haas M. (2012). So many teams, so little time: time allocation matters in geographically dispersed teams. *J. Organ. Behav.* 33 316–341. 10.1002/job.777

[B32] De Dreu C. K. W. Weingart L. R. (2003). Task versus relationship conflict, team performance, and team member satisfaction: a meta-analysis. *J. Appl. Psychol.* 88 741–774. 12940412 10.1037/0021-9010.88.4.741

[B33] Devine D. J. (2002). A review and integration of classification systems relevant to teams in organizations. *Group Dyn. Theory Res. Pract.* 6 291–310. 10.1037//1089-2699.6.4.291

[B34] Devine D. J. Clayton L. D. Philips J. L. Dunford B. B. Melner S. B. (1999). Teams in organizations: prevalence, characteristics, and effectiveness. *Small Group Res.* 30 678–711. 10.1177/104649649903000602

[B35] Dineen B. R. Noe R. A. (2003). “The impact of team fluidity and its implications for human research management research and practice,” in *Research in Personnel and Human Resources Management* eds Martocchio J. Ferris G. (Bingley: Emerald Group Publishing) 1–38.

[B36] Driskell J. E. Salas E. Driskell T. (2018). Foundations of teamwork and collaboration. *Am. Psychol.* 73 334–348. 10.1037/amp0000241 29792452

[B37] Driskell T. Salas E. Driskell J. E. (2018). Teams in extreme environments: alterations in team development and teamwork. *Hum. Resour. Manag. Rev.* 28 434–449. 10.1016/j.hrmr.2017.01.002

[B38] Dube L. (2014). Exploring how IT professionals experience role transitions at the end of successful projects. *J. Manag. Inf. Syst.* 31 17–46. 10.2753/mis0742-1222310102

[B39] Edmondson A. C. (2012). *Teaming: How Organizations Learn, Innovate, and Compete in the Knowledge Economy.* San Francisco, CA: Jossey-Bass.

[B40] Edmonson A. C. Harvey J.-F. (2018). Cross-boundary teaming for innovation: integrating research on teams and knowledge in organizations. *Hum. Resour. Manag. Rev.* 28 347–360. 10.1016/j.hrmr.2017.03.002

[B41] Espinosa J. A. Cummings J. N. Pickering C. (2012). Time separation, coordination, and performance in technical teams. *IEEE Trans. Eng. Manag.* 59 91–103. 10.1109/tem.2011.2126579

[B42] Feitosa J. Grossman R. Salazar M. (2018). Debunking key assumptions about teams: the role of culture. *Am. Psychol.* 73 376–389. 10.1037/amp0000256 29792455

[B43] Francis D. Young D. (1970). *Improving Work Groups: A Practical Manual for Teambuilding.* La Jolla, CA: University Associates.

[B44] Frick S. E. Fletcher K. A. Rasay P. S. Bedwell W. L. (2018). Understanding team maladaptation through the lens of the four R’s of adaptation. *Hum. Resour. Manag. Rev.* 28 411–422. 10.1016/j.hrmr.2017.08.005

[B45] Fritz C. Yankelevich M. Zarubin A. Barger P. (2010). Happy, healthy, and productive: the role of detachment from work during nonwork time. *J. Appl. Psychol.* 95 977–983. 10.1037/a0019462 20836591

[B46] Gilson L. L. Maynard M. T. Young N. C. J. Vartianinen M. Hakonen M. (2015). virtual teams research: 10 years, 10 themes, and 10 opportunities. *J. Manag.* 41 1313–1337. 10.1177/0149206314559946

[B47] Gladstein D. L. (1984). Groups in context: a model of task group effectiveness. *Adm. Sci. Q.* 29 499–517. 10.2307/2392936

[B48] Gonzalez V. Mark G. (2004). “Constant, constant, multi-tasking craziness: managing multiple working spheres,” in *Paper Presented at the SIGCHI Conference on Human Factors in Computing Systems* New York, NY.

[B49] Graesser A. C. Fiore S. M. Greiff S. Andrews-Todd J. Foltz P. W. Hesse F. W. (2018). Advancing the science of collaborative problem solving. *Psychol. Sci. Public Interest* 19 59–92. 30497346 10.1177/1529100618808244

[B50] Gully S. M. Devine D. J. Whitney D. J. (1995). A meta-analysis of cohesion and performance: effects of level of analysis and task interdependence. *Small Group Res.* 26 497–520. 10.1177/1046496495264003

[B51] Gully S. M. Incalcaterra K. A. Joshi A. Beaubien J. M. (2002). A meta-analysis of team-efficacy, potency, and performance: interdependence and level of analysis and moderators of observed relationships. *J. Appl. Psychol.* 87 819–832. 10.1037/0021-9010.87.5.819 12395807

[B52] Guzzo R. A. Dickson M. W. (1996). Teams in organizations: recent research on performance and effectiveness. *Annu. Rev. Psychol.* 47 307–338. 10.1146/annurev.psych.47.1.307 15012484

[B53] Hackman J. R. (1987). “The design of work teams,” in *Handbook of Organizational Behavior* ed. Lorsch J. (Englewood Cliffs, NJ: Prentice-Hall) 315–342.

[B54] Hackman J. R. (1990). *Groups that Work (and those that don’t).* San Francisco, CA: Jossey-Bass.

[B55] Hackman J. R. (2002). *Leading Teams. Setting the Stage for Great Performances.* Brighton, MA: Harvard Business School Press.

[B56] Hackman J. R. (2012). From causes to conditions in group research. *J. Organ. Behav.* 33 428–444. 10.1002/job.1774

[B57] Harrison D. A. Humphrey S. E. (2010). Designing for diversity or diversity for design? Tasks, interdependence, and within-unit differences at work. *J. Organ. Behav.* 31 328–337. 10.1002/job.608

[B58] Hirst G. (2009). Effects of membership change on open discussion and team performance: the moderating role of team tenure. *Eur. J. Work Organ. Psychol.* 18 231–249. 10.1080/13594320802394202

[B59] Hollenbeck J. R. Beersma B. Schouten M. E. (2012). Beyond team types and taxonomies: a dimensional scaling conceptualization for team description. *Acad. Manag. Rev.* 37 82–106. 10.5465/armr.2010.0181

[B60] Hollenbeck J. R. Ilgen D. R. Sego D. J. Hedlund J. Major D. A. Phillips J. (1995). Multilevel theory of team decision making: decision performance in teams incorporating distributed expertise. *J. Appl. Psychol.* 80 292–316. 10.1037/0021-9010.80.2.292

[B61] Hu J. Liden R. C. (2011). Antecedents of team potency and team effectiveness: an examination of goal and process clarity and servant leadership. *J. Appl. Psychol.* 96 851–862. 10.1037/a0022465 21319877

[B62] Ilgen D. R. Hollenbeck J. R. Johnson M. Jundt D. (2005). Teams in organizations: from input-process-output models to IMOI models. *Annu. Rev. Psychol.* 56 517–543. 10.1146/annurev.psych.56.091103.070250 15709945

[B63] Katzenbach J. R. Smith D. K. (1998). *The Wisdom of Teams.* Berkshire: McGraw-Hill.

[B64] Kazemak E. Albert B. (1988). Learning the secret to teamwork. *Healthc. Financ. Manag.* 42 108–110. 10288884

[B65] Klein H. J. Mulvey P. W. (1995). Two investigations of the relationships among group goals, goal commitment, cohesion, and performance. *Organ. Behav. Hum. Dec. Process.* 61 44–53. 10.1006/obhd.1995.1004

[B66] Klein K. Kozlowski S. W. J. (2000). *Multilevel theory, Research and Methods in Organization.* San Francisco, CA: Jossey-Bass.

[B67] Kozlowski S. W. J. Bell B. S. (2003). “Work groups and teams in organizations,” in *Comprehensive Handbook of Psychology: Industrial and Organizational Psychology* Vol. 12 eds Borman W. Ilgen D. Klimoski R. (New York, NY: Wiley) 333–375.

[B68] Kozlowski S. W. J. Gully S. M. Nason E. R. Smith E. M. (1999). “Developing adaptive teams: a theory of compilation and performance across levels and time,” in *The Changing Nature of Work Performance: Implication for Staffing, Personnel Actions and Development* eds Ilgen D. R. Pulakos E. D. (San Francisco, CA: Jossey Bass) 240–292.

[B69] Kozlowski S. W. J. Ilgen D. R. (2006). Enhancing the effectiveness of work groups and teams. *Psychol. Sci. Public Interest* 7 77–124. 10.1111/j.1529-1006.2006.00030.x 26158912

[B70] Lacerenza C. N. Marlow S. M. Tannenbaum S. I. Salas E. (2018). Team development interventions: evidence-based approaches for improving teamwork. *Am. Psychol.* 73 517–531. 10.1037/amp0000295 29792465

[B71] Landrum S. (2017). *Millennials Aren’t Afraid to Change Jobs, and Here’s why. Forbes.* Available at: https://www.forbes.com/sites/sarahlandrum/2017/11/10/millennials-arent-afraid-to-change-jobs-and-heres-why/#2688a85719a5 (accessed November 10 2017).

[B72] Lanza P. (1985). Team appraisals. *Pers. J.* 64 47–51.

[B73] LePine J. A. (2005). Adaptation of teams in response to unforeseen change: effects of goal difficulty and team composition in terms of cognitive ability and goal orientation. *J. Appl. Psychol.* 90 1153–1167. 10.1037/0021-9010.90.6.1153 16316271

[B74] LePine J. A. Piccolo R. F. Jackson C. L. Mathieu J. E. Saul J. R. (2008). A meta-analysis of teamwork processes: tests of a multidimensional model and relationships with team effectiveness criteria. *Pers. Psychol.* 61 273–307. 10.1111/j.1744-6570.2008.00114.x

[B75] Lewis K. Belliveau M. Herndon B. Keller J. (2007). Group cognition, membership change, and performance: investigating the benefits and detriments of collective knowledge. *Organ. Behav. Hum. Dec. Process.* 103 159–178. 10.1016/j.obhdp.2007.01.005

[B76] Malone T. W. Crowston K. (1994). The interdisciplinary study of coordination. *ACM Comput. Surv.* 26 87–119. 10.1145/174666.174668

[B77] Marks M. A. Mathieu J. E. Zaccaro S. J. (2001). A temporally based framework and taxonomy of team processes. *Acad. Manag. Rev.* 26 356–376. 10.5465/amr.2001.4845785

[B78] Martin A. Bal V. (2006). *The State of Teams.* Greensboro, NC: Center for Creative Leadership.

[B79] Mathieu J. Maynard M. T. Rapp T. Gilson L. (2008). Team effectiveness 1997-2007: a review of recent advancements and a glimpse into the future. *J. Manag.* 34 410–476. 10.1177/0149206308316061

[B80] Mathieu J. E. Hollenbeck J. R. van Knippenberg D. Ilgen D. R. (2017). A century of work teams in the Journal of Applied Psychology. *J. Appl. Psychol.* 102 452–467. 10.1037/apl0000128 28150984

[B81] Mathieu J. E. Tannenbaum S. I. Donsbach J. S. Alliger G. A. (2014). A review and integration of team composition models: moving toward a dynamic and temporal framework. *J. Manag.* 40 130–160. 10.1177/0149206313503014

[B82] McGrath J. E. (1964). *Social Psychology: A Brief Introduction.* New York, NY: Holt, Rinehart, & Winston.

[B83] McGrath J. E. Arrow H. Berdahl J. L. (2000). The study of groups: past, present, and future. *Pers. Soc. Psychol. Rev.* 4 95–105. 10.1207/S15327957PSPR0401_8

[B84] Microsoft (2015). *Attention Spans.* Canada: Consumer Insights, Microsoft Canada.

[B85] Miller L. K. Hamblin R. L. (1963). Interdependence, differential rewarding, and productivity. *Am. Sociol. Rev.* 28 768–778. 10.2307/2089914

[B86] Mitchell T. R. Silver W. S. (1990). Individual and group goals when workers are interdependent: effects on task strategies and performance. *J. Appl. Psychol.* 75 185–193. 10.1037//0021-9010.75.2.185

[B87] Morgan B. B. Jr. Salas E. Glickman A. S. (1993). An analysis of team evolution and maturation. *J. Gen. Psychol.* 120 277–291. 10.1080/00221309.1993.9711148

[B88] Mortensen M. Haas M. R. (2016). *Rethinking Team Boundaries.* Available at: https://papers.ssrn.com/sol3/papers.cfm?abstract_id=2841150 (accessed September 21 2016).

[B89] Murase T. Doty D. Wax A. DeChurch L. Contractor N. S. (2012). Teams are changing: time to “think networks”. *Ind. Organ. Psychol.* 5 41–44. 10.1111/j.1754-9434.2011.01402.x

[B90] Okhuysen G. A. Bechky B. A. (2009). 10 coordination in organization: an integrative perspective. *Acad. Manag. Ann.* 3 463–502. 10.5465/19416520903047533

[B91] O’Leary M. B. Woolley A. W. Mortensen M. (2011). “Multiteam membership in relation to multiteam systems,” in *Multiteam Systems: An Organization form for Dynamic and Complex Environments* eds Zaccaro S. J. Marks M. A. DeChurch L. A. (New York, NY: Routledge) 141–172.

[B92] Rasmussen T. H. Jeppesen H. J. (2006). Teamwork and associated psychological factors: a review. *Work Stress* 20 105–128. 10.1080/02678370600920262

[B93] Richardson J. (2010). *An Investigation of the Prevalence and Measurement of Teams in Organisations: The Development and Validation of the Real Team Scale.* Doctoral dissertation, Aston University Birmingham.

[B94] Robert Half (2018). *Does Job Hopping Help or Hurt Your Career?* Available at: http://rh-us.mediaroom.com/2018-04-05-Does-Job-Hopping-Help-Or-Hurt-Your-Career?utm_campaign=Press_Release&utm_medium=Link&utm_source=Press_Release (accessed April 5 2018).

[B95] Rousseau V. Aubé C. Savoie A. (2006). Teamwork behaviors: a review and an integration of Frameworks. *Small Group Res.* 37 540–570. 10.1136/bmjopen-2015-007685 25652806 PMC4322213

[B96] Saavedra R. Earley P. Van Dyne L. (1993). Complex interdependence in task-performing groups. *J. Appl. Psychol.* 78 61–72. 10.1037/0021-9010.78.1.61

[B97] Salas E. Benishek L. E. Coultas C. Dietz A. Grossman R. Lazzara E. H. (2015). *Team Training Essentials: A Research-Based Guide.* New York, NY: Routledge.

[B98] Salas E. Dickinson T. L. Converse S. A. Tannenbaum S. I. (1992). “Toward an understanding of team performance and training,” in *Teams: Their Training and Performance* eds Swezey R. W. Salas E. (Norwood, NJ: Ablex) 3–29.

[B99] Salas E. Shuffler M. L. Thayer A. L. Bedwell W. L. Lazzara E. H. (2011). Understanding and improving teamwork in organizations: a scientifically based practical guide. *Hum. Resour. Manag.* 54 599–622. 10.1002/hrm.21628

[B100] Salas E. Sims D. E. Burke C. S. (2005). Is there a “big five” in teamwork? *Small Group Res.* 36 555–599. 10.1177/1046496405277134 20046712

[B101] Salas E. Stagl K. C. Burke C. S. Goodwin G. F. (2007). “Fostering team effectiveness in organizations: toward an integrative theoretical framework of team performance,” in *Modeling Complex Systems: Motivation, Cognition and Social Processes* eds Dienstbier R. A. Shuart J. W. Spaulding W. Poland J. (Lincoln, NE: University of Nebraska Press) 185–243. 17682335

[B102] Salas E. Tannenbaum S. I. Kraiger K. Smith-Jentsch K. A. (2012). The science of training and development in organizations: what matters in practice. *Psychol. Sci. Public Interest* 13 74–101. 10.1177/1529100612436661 26173283

[B103] Savelsbergh C. M. J. H. Poell R. F. van der Heijden B. I. J. M. (2015). Does team stability mediate the relationship between leadership and team learning? An empirical study among Dutch project teams. *Int. J. Proj. Manag.* 33 406–418. 10.1016/j.ijproman.2014.08.008

[B104] Schippers M. C. Hartog D. N. D. Koopman P. L. (2007). Reflexivity in teams: a measure and correlates. *Appl. Psychol.* 56 189–211. 10.1111/j.1464-0597.2006.00250.x

[B105] Shank P. (2012). *Smart Companies Support informal Learning.* Available at: https://www.elearningguild.com/insights/index.cfm?id=159&action=viewonly&utm_campaign=research-inf12&utm_medium=link&utm_source=lsmag&_ga=2.249953734.1734585252.1543552949-1329011644.1543552949 (accessed August 16 2012).

[B106] Shea G. P. Guzzo R. A. (1987). “Groups as human resources,” in *Research in Personnel and Human Resources Management* Vol. 5 eds Rowland K. M. Ferris G. R. (Greenwich, CT: JAI Press) 323–356.

[B107] Shuffler M. L. Carter D. R. (2018). Teamwork situated in multiteam systems: key lessons learned and future opportunities. *Am. Psychol.* 73 390–406. 10.1037/amp0000322 29792456

[B108] Summers J. K. Humphrey S. E. Ferris G. R. (2012). Team member change, flux in coordination, and performance: effects of strategic core roles, information transfer, and cognitive ability. *Acad. Manag. J.* 55 314–338. 10.5465/amj.2010.0175

[B109] Sundstrom E. De Meuse K. P. Futrell D. (1990). Work teams: applications and effectiveness. *Am. Psychol.* 45 120–133. 10.1037//0003-066x.45.2.120

[B110] Tannenbaum S. I. Mathieu J. E. Salas E. Cohen D. (2012). Teams are changing: are research and practice evolving fast enough. *Ind. Organ. Psychol.* 5 2–24. 10.1111/j.1754-9434.2011.01396.x 27606397

[B111] van de Brake H. J. Walter F. Rink F. A. Essens P. J. M. D. van der Vegt G. S. (2018). The dynamic relationship between multiple team membership and individual job performance in knowledge-intensive work. *J. Organ. Behav.* 39 1219–1231. 10.1002/job.2260 30555212 PMC6282989

[B112] Van der vegt G. S. Emans B. J. M. van de vliert E. (2001). Patterns of interdependence in work teams: a two-level investigation of the relations with job and team satisfaction. *Pers. Psychol.* 54 51–69. 10.1111/j.1744-6570.2001.tb00085.x

[B113] Wageman R. (1995). Interdependence and group effectiveness. *Adm. Sci. Q.* 40 145–180. 10.2307/2393703

[B114] Wageman R. Gardner H. Mortensen M. (2012). The changing ecology of teams: new directions for teams research. *J. Organ. Behav.* 33 301–315. 10.1002/job.1775

[B115] Wageman R. Hackman J. R. Lehman E. (2005). Team diagnostic survey: development of an instrument. *J. Appl. Behav. Sci.* 41 373–398. 10.1177/0021886305281984

[B116] Weingart L. R. (1992). Impact of group goals, task component complexity, effort, and planning on group performance. *J. Appl. Psychol.* 77 682–693. 10.1037/0021-9010.77.5.682

[B117] Weldon E. Weingart L. R. (1993). Group goals and group performance. *Br. J. Soc. Psychol.* 32 307–334. 10.1111/j.2044-8309.1993.tb01003.x

[B118] West M. A. (2004). *Effective Teamwork: Practical Lessons from Organizational Research.* Oxford: Blackwell.

[B119] West M. A. Borrill C. S. Unsworth K. L. (1998). “Team effectiveness in organisations,” in *International Review of Industrial and Organisational Psychology* eds Cooper C. L. Robertson I. T. (Chichester: Wiley) 1–48.

[B120] West M. A. Lyubovnikova J. (2012). Real teams or pseudo teams: the changing landscape needs a better map. *Ind. Organ. Psychol.* 5 25–55.

[B121] Wildman J. L. Thayer A. L. Rosen M. A. Salas E. Mathieu J. E. Rayne S. R. (2011). Task types and team-level attributes: synthesis of team classification literature. *Hum. Resour. Dev. Rev.* 11 37–129.

[B122] Zander A. (1977). *Groups at Work.* San Francisco, CA: Jossey-Bass.

[B123] Ziller R. C. (1965). Toward a theory of open and closed groups. *Psychol. Bull.* 64 164–182. 10.1037/h0022390 14343396

